# A Painful Situation Both for Patient and Practitioner, and the Advantage of Presence of Mind to the Latter

**Published:** 1854-07

**Authors:** J. L. Levison


### ARTICLE V.

### *A Painful Situation both for Patient and Practitioner, and the Advantage of Presence of Mind to the latter.* By J. L. Levison, D. D. S., &c.

Mr. H———, a nice old gentleman, about seventy-five years old, applied for my professional services, and I incidentally noticed at that time, that he had strong symptoms of disease of the heart.

Subsequently, having had an accident with his bone lower jaw, he called upon me for another, which he specified must be ready at such an hour, and on a particular day. The promise was made, and punctually to the moment, his carriage drove to my door, and soon my servant showed him into my surgery.

It may be remarked, that he had been a free liver, enjoying life, as it is called, and which in his early days, it is probable, that he would not do what would have been deemed a great disgrace—sherk his bottle; but which now, by a change of custom for better, would have been regarded the better proof of an educated man. He was, however, a very nice, urbane, and cultivated gentleman, about the middle height in stature—with light blue eyes, a florid face, and of a sanguineous temperament; and as he entered, having more closely observed him and his mode of breathing, it was evident that he was affected with *angina pectoris*. After an almost automaton bow of recognition, I pointed to my operating chair, and he sat down without either of us having spoken a word. But as I turned my back to him, in order to open my mahogany work-board, I merely said, "You are very punctual, sir!" when immediately after I heard a curious gurgling sound, and turning round to ascertain what it could be, I saw my patient with his head thrown backwards—his face livid—his eyes fixed, and his lips open and motionless, as if he was either in *articulo mortis*, or had actually already given up the ghost! My first act was to feel his pulse, but he did not seem to have one, and yet I could not believe him to be dead. I therefore threw open the window, and dashed some cold water in his face, and watched the result with breathless anxiety. Soon, to my great satisfaction, I saw him move. He then opened his eyes with a peculiar stare, like one suddenly recovered from a fit—then he endeavored to speak, but his tongue moved heavily, and there was a thickness and indistinctness in his words, in the manner of one partially apoplectic. Whilst I was perspiring at every pore, he pointed to his mouth, and seemed impatient and somewhat annoyed that I did not proceed. Having tried the fit—which was very good—yet I was so anxious lest he should relapse, that I urged him to call another day, and would willingly have foregone the receipt of the fee to have got him safely out of my house. But the good old man, by gesticulation and his "unknown tongue," seemed to insist on my keeping my bond, so I put in the jaw. It fitted him admirably well. He felt in his pocket, muttered something, then felt again, as if annoyed at his disappointment. Interpreting these actions to the fact that he had forgotten his purse, I said, "never mind the money, sir;" at which he said something which I could not understand. Again his head fell backwards, and his face assumed a darker hue, and again his spirit seemed as if it had departed.

I had recourse to similar treatment, though it appeared a forlorn hope. Still I persevered, and placed some strong ammoniacal salts to his notrils, and after a vast amount of labor and anxiety, he once more, to my great relief, breathed again. And as he recommenced seeking for his purse, I repeated, "Pray, never mind the money, you are very ill, do let me beg of you to return to your residence?" "No! no! no!" said the worthy man—"some paper! some paper!" I gave him paper, under the notion that it would be a means of expediting his departure, and so it did; for he wrote a few hieroglyphical signs for a check, and then I induced him to take my arm, and as quickly as my humanity permitted, I got him safely to the street door, which my boy opened, and all assisted the coachman to get the patient into his carriage. He was driven to his temporary home, (as he was a visitor at Brighton,) and died a few hours afterwards, having, however, sent the fee immediately on his return.

What may be called *presence of mind* in this case, might be rendered thus: "That though greatly agitated at the prospect and annoyance of a coroner's inquest at my own residence—these thoughts mocked me, inducing some agitation—at the same time being cognizant of the fact, that unless I could stimulate him by the means used, so as to rouse his brain and excite the heart's action, that there was not the least chance of his recovery; and it was, therefore, the cool and deliberate manner which carried into effect the decision of the intellect, and the firmness of purpose by which I persisted in my operations, (varying the means and their application according to the particular exigency;) it was these combined, (the intellectual powers and firmness,) which constitutes what men designate "*presence of mind!*" and which is easily distinguished from those mere impulsive acts under some difficulty, as much as is the difference of a practitioner acting from sound theoretical views, and one who is merely guided by some empirical treatment. The latter may succeed—the former *must* do so; and therefore, in any unexpected difficulty, the question with myself has ever been—what is this? what has caused it? how shall it be treated? And when satisfied on these points for all practical purposes, I act promptly, and recommend all "to do so likewise."

